# The strength of weak bonds: Using a novel ecosystem approach to promote public sector scaling of innovations in resource limited settings

**DOI:** 10.3389/fpubh.2022.1027652

**Published:** 2022-10-24

**Authors:** Francis Wafula, Thomas Feeny, Open Phences, Irene Khayoni

**Affiliations:** ^1^Open Phences Hub, Strathmore University Business School, Nairobi, Kenya; ^2^Innovation Practice, Results for Development, Washington, DC, United States

**Keywords:** innovation, technology, ecosystem-building, partnership, Africa

## Introduction

Recent years have seen unprecedented growth in the scale and scope of tech innovations across sub-Saharan Africa and other low- and middle-income countries (LMICs) (1, 2). This was further accelerated by the COVID-19 pandemic, which disrupted the traditional forms of health service delivery, forcing providers to adopt “non-physical” ways of providing care (3). The general view is that we are unlikely to revert to the pre-pandemic state and that new technologies will continue to emerge across different facets of healthcare. We have seen a wide variety of tech innovations, ranging from tools for communicating to communities, electronic health records and telemedicine to more advanced applications for creating intelligent systems such as machine learning and artificial intelligence (2).

At present, most innovations scale through the market, rarely penetrating public healthcare systems. This contributes to inequity by locking out vulnerable groups that seek care from public facilities (4). This is unusual on the one hand because government systems offer a “ready-made” pathway to scale that may help them reach millions of people quickly while having to devote less time and effort to generating demand in a highly competitive environment. However, widespread perceptions of government as less innovative and the notion of public health systems being slow and resistant to change have discouraged many innovators from considering the public sector as a feasible or attractive scaling pathway. Other reasons for low public sector scaling (PSS) of potentially useful innovations include a lack of awareness within government around innovations that exist, mistrust between public and private sectors hindering their collaboration for scale-up, inadequate inclusion of innovators and other non-state actors in planning and low emphasis on sustainability, partly caused by overreliance on aid. The gradual transition away from aid dependency is therefore a watershed moment, presenting LMIC governments with the opportunity to pursue alternative priority setting and investment mechanisms that make better use of local ecosystem actors, including innovators.

While there is growing evidence showing that scaling up digital health innovation within the public sector could accelerate progress toward universal health coverage (2), there is still very little research and analysis that has specifically set out to explore how PSS scaling could be achieved, more so, using the ecosystem approach. That said, a recent analysis linked the use of the co-creation approach to improved adoption of digital health innovations (5).

**Figure 1:**
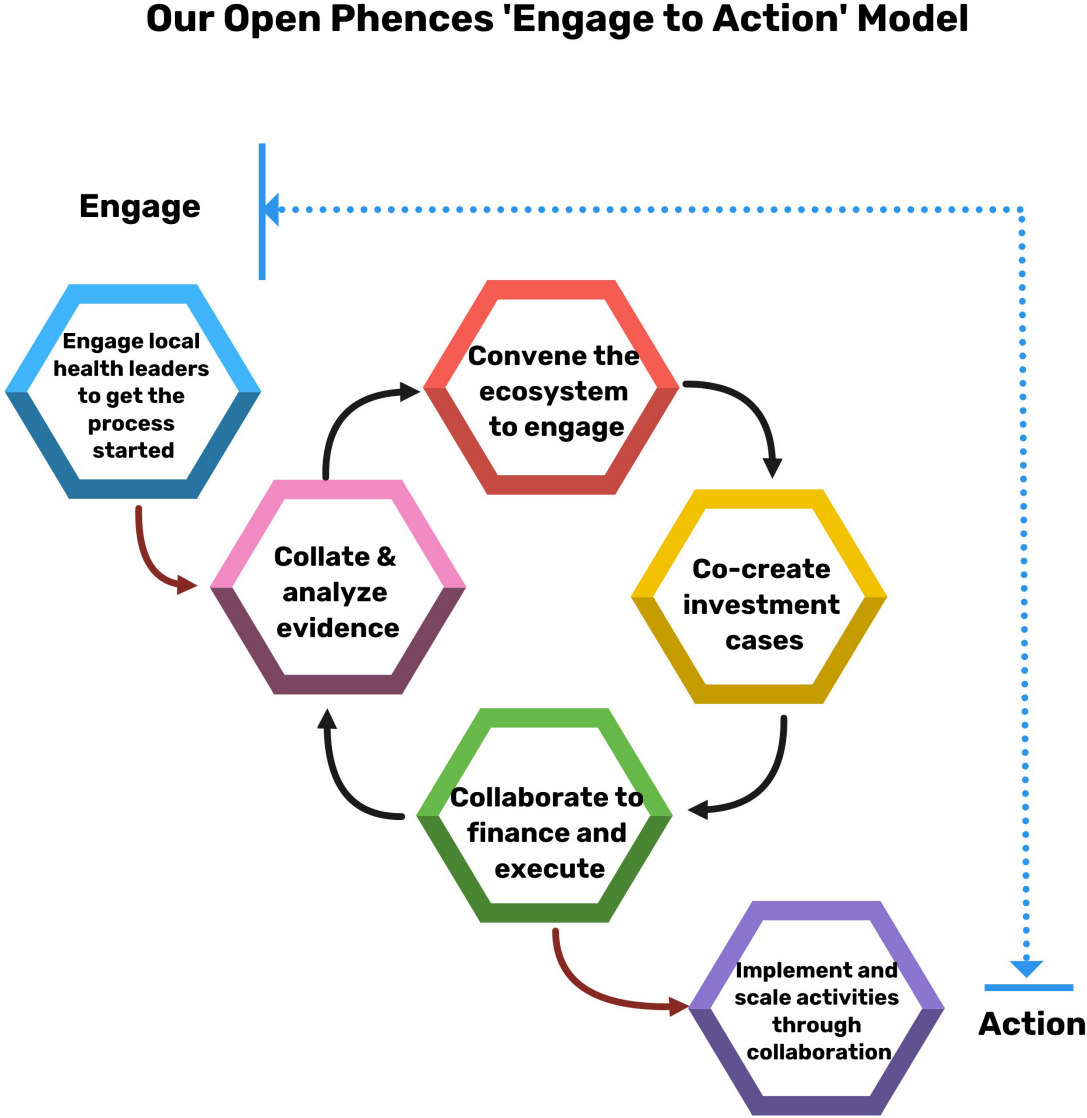
The Open Phences “Engage to Action” model.

In this commentary, we look at how an ecosystem approach could be used to create value prepositions that support the shift toward PSS. We present the Open Phences “Engage to Action” model (Figure 1) that entails facilitating ecosystem actors to identify genuine need and express demand for the right innovations. Our title borrows heavily from Nick Granovetter's “Strength of Weak Ties,” theory, which posits that “weak ties” (meaning individuals that are loosely connected to a person) are more likely to provide new/more useful information when the person is in need, compared to the “strong ties,” who are likely to be individuals with similar information and characteristics as the person in question. We build on this line of thought, arguing that public sector scaling of private innovations could massively strengthen health services through injecting new ideas and interventions, despite the “weak bonds” that typically link the public and private health sectors across many SSA countries.

## The ecosystem building, planning, and investment approach

Open Phences is a “Think and Do Hub” that works to democratize public private collaborations in health. The Hub's “Engage to Action” model (Figure 1) proposes a unique approach to ecosystem building, joint priority-setting and planning, and investing based on shared goals. The aim is to create a shift from the push-model (where innovators compete to sell innovations to a usually disinterested ecosystem) to the “pull-model” (where a cohesive and informed ecosystem defines priorities and demands the right innovations).

Starting December 2021 with seed funding from the UK Foreign, Commonwealth and Development Office (FCDO), Open Phences is piloting the Model's potential to improve RMNCAH services in four Kenyan counties of Homa Bay, Kisumu, Kiambu and Trans Nzoia (RMNCAH stands for reproductive, maternal, neonatal, child and adolescent health). For every county, four key steps are being followed namely, (i) understanding the RMNCAH situation and mapping existing innovators and innovations, (ii) supporting county-led RMNCAH ecosystem building and cohesion activities, (iii) facilitating co-creation workshops to identify priority challenges and develop suitable investment cases, and (iv) identifying public and private investors and funders to fill the funding gaps in the investment cases and get a suitable return (e.g., social impact, equity, or a fair commercial return). Central to all these is the inclusion of the most suitable tech innovations in the county plans, which increases the probability of scaling them through the public health sector and with funding from Treasury and the counties.

The first step entails understanding the RMNCAH situation (the need/demand) and existing innovations (the supply), which provides the information needed for ecosystem actors to engage in meaningful discussion downstream. The assessment considers tech and non-tech solutions that are contributing directly and indirectly toward improving RMNCAH. The first step ends with each county having a RMNCAH and innovations situation report.

The second step involves mapping ecosystem actors and helping to organize them into associations with elected representatives for subsequent engagement with government. Here, we borrowed from the World Bank Toolkit for mapping stakeholder for public private dialogue, which details crucial steps, including ways of identifying less visible actors and understanding motivations (6). The ecosystem actors are brought together to discuss the RMNCAH report, share experiences, and agree on priority challenges affecting their communities. It is at this stage that the role of innovations is discussed in detail. The second step ends with each county having a RMNCAH priority investment areas report that highlights the most pressing challenges and potential solutions. Ecosystem actors involved include public and private sector representatives for different areas, including medical service providers, pharmacy, diagnostics, nutrition services, childhood education and agriculture among others. Also included are health financing institutions and community representatives selected to ensure gender, equity and social inclusion.

In the third stage, we support the ecosystem actors to refine the priority investment areas and develop costed RMNCAH investment cases, with funding gaps highlighted. Here, we borrow from the widely tested and validated Global Financing Facility (GFF) approach to developing investment cases for maternal and child health across resource constrained settings (7).

The final step involves two sets of activities: embedding the investment cases into the county planning processes to benefit from Treasury allocation in future funding cycles; and connecting county leadership to other potential funders to help fill the funding gaps. Partners may include blended financing or impact investors, traditional debt and equity investors, as well as other untapped sources like local high net worth individuals who may be persuaded to lend support against well-defined investment cases.

## Discussion—What opportunities does the novel approach present?

While we are still testing the model across the four counties (now entering step 3 above), we have already made some notable observations.

First, we note that the approach allows elaboration of priority challenges and RMNCAH gaps that represent genuine inadequacies. For instance, in cases where county managers would have asked for funds to establish an operating theater for obstetric emergencies, they are now asking for a much lower investment—e.g., by contracting theater space from idle private facilities within the county, a solution unearthed through the engagements across the ecosystem. We are seeing new opportunities emerge, including the potential to scale innovative tele-radiology and tele-pharmacy services through public channels. We are also beginning to see stronger emphasis on community services, with proposals to incorporate tech innovations to expand the service offering, all proposed by the actors during co-creation. The value of creating systems that encourage prioritization of digital solutions is something that has been emphasized in previous discussions (8).

Second, we are seeing a more democratic, level playing field for innovators in terms of how they are engaged and assessed by government actors. The Model is helping to create a fair platform for innovators to present their products, and for providers to share experiences with the use of the innovations in the local environment. Prior to that, the innovators' market was highly fragmented, with low visibility for decisionmakers and little opportunity for innovators to showcase their work in comparison to others. This results in low public sector uptake, and whenever innovations find their way to public systems, it is usually because those behind them have some leverage with decision makers. The risk here is that the products taken up are not selected on the basis of the evidence and may be neither the most innovative nor impactful, in the process creating an inequitable procurement environment that stifles innovation and healthy competition. Our proposed approach may also contribute to higher scrutiny and use of proper methods to evaluate digital health innovations, crowding out poorly designed ones and building trust among users (9). What we are seeing is the ecosystem approach building trust among actors and allowing a more open discussion on the most appropriate choices to make. This injects transparency in the process, reducing risk to all concerned and making it easier to justify adopting and scaling the innovation. Credibility and legitimacy have been shown to be important contributors to successful scaling of innovations (10).

Finally, we are starting to see the approach creating clearer pathways to scale through their inclusion in county plans. In so doing, counties can competitively award contracts to innovators with the most suitable solutions, and have the costs covered through their core budgets. This is crucial, considering the government is the largest single payer and provider of healthcare services in most LMICs. Our hope is that these currently fragmented instances of public sector scaling become the new norm, part of a strong and sustainable market in which demand and supply are matched efficiently and appropriately through a sustainable and credible vehicle. We believe that this is the most sustainable pathway for generating impact at scale through tech innovations in health. At the same time, establishment of such pathways will trigger more innovation, as entrepreneurs aim to satisfy the expressed demand. One lesson we got from the height of the COVID-19 pandemic, was the fact that whenever need is expressed at scale, traditional barriers to uptake of innovations can be suspended, including regulatory hoops and low motivation to change/adopt technology (5).

To share the learning from our model and further support the adoption of public sector scaling approaches, Open Phences are now proudly working with others across East Africa as part of a new Public Sector Scaling Action Lab facilitated by Results for Development with support from Grand Challenges Canada. The Lab comprises of a group of healthcare champions who are researching, designing and testing new partnership approaches that have the potential to improve public sector sourcing and scaling of the most suitable innovations for public good.

A key strength of our opinion piece stems from the fact that we are drawing lessons from a real-life project in a low-resource setting. In addition, we believe that the approach proposed is sector agnostic, and could strengthen other areas like education, water and environment, and social services. However, the fact that the project is still ongoing and hasn't been evaluated presents the main point of weakness in our view. It is possible that new insights may emerge that change how we have presented our thoughts. Further, our approach feels suited to a decentralized country context, where crucial decisions that touch on priority-setting and resource allocation can be made through local ecosystem building effort. Strongly centralized economies may have higher diversity in ecosystem formation and interests, possibly needing a modified approach. That said, there is value in sharing these kinds of experiences early, especially in the context of a growing pipeline of innovations that are not thinking about public sector scaling as an option for growth, equity, and sustainability.

## Conclusion

The public private sector discourse has been excessively dichotomized, creating a schism, a black and white situation. Yet, we believe that there is a lot of gray in between—not least the opportunity to generate faster and greater impact through the scale-up of innovation within the public sector. This can be achieved sustainably through ecosystem-wide participation in planning, resource allocation and investment, but this requires a strategic approach such as the one presented here. To paraphrase Henry Ford, nothing is particularly hard if you divide it into small tasks. There is value in taking a stepwise approach that includes building the ecosystem, creating mutual trust, identifying common and high priority challenges, co-creating solutions and investment cases, and finally, working together to identify gaps in resources and bring on board investors and partners. This is a faster and more pragmatic alternative to structuring complex longwinded public private partnership contracts.

## Open Phences

Members of Open Phences are Lyndon Marani, Irene Khayoni, Noelle Orata, Brenda Bunyasi, Annette Murunga, Elizabeth Gitau, Eric Tama, Cornelius Kiptoo, Paul Waswa, Muriithi Njogu, and Peter Nguhiu.

## Author contributions

FW conceived and wrote the first draft of the opinion piece. TF contributed to the conception and drafting. The Open Phences team members are implementing the project on which the opinions shared are partially based. All authors contributed to the article and approved the submitted version.

## Funding

Seed funding was provided by the UK Foreign, Commonwealth and Development Office (FCDO) through the RISA funding stream.

## Conflict of interest

Author FW is a member of the Open Phences, the team that is piloting the model that has been presented and discussed in this paper. The remaining authors declare that the research was conducted in the absence of any commercial or financial relationships that could be construed as a potential conflict of interest.

## Publisher's note

All claims expressed in this article are solely those of the authors and do not necessarily represent those of their affiliated organizations, or those of the publisher, the editors and the reviewers. Any product that may be evaluated in this article, or claim that may be made by its manufacturer, is not guaranteed or endorsed by the publisher.
